# AATF/Che-1 RNA polymerase II binding protein overexpression reduces the anti-tumor NK-cell cytotoxicity through activating receptors modulation

**DOI:** 10.3389/fimmu.2023.1191908

**Published:** 2023-06-26

**Authors:** Matteo Caforio, Nicola Tumino, Cristina Sorino, Isabella Manni, Stefano Di Giovenale, Giulia Piaggio, Simona Iezzi, Georgios Strimpakos, Elisabetta Mattei, Lorenzo Moretta, M. Fanciulli, Paola Vacca, Franco Locatelli, Valentina Folgiero

**Affiliations:** ^1^ Department of Pediatric Hematology and Oncology, Cell and Gene Therapy, Bambino Gesù Children’s Hospital, Istituto di Ricerca e Cura a Carattere Scientifico (IRCCS), Rome, Italy; ^2^ Immunology Research Area, Innate Lymphoid Cells Unit, Bambino Gesù Children’s Hospital Istituto di Ricerca e Cura a Carattere Scientifico (IRCCS), Rome, Italy; ^3^ Stabilimento Allevamento Fornitore e Utilizzatore (SAFU) Laboratory, Department of Research, Advanced Diagnostic, Technological Innovation, Regina Elena National Cancer Institute Istituto di Ricerca e Cura a Carattere Scientifico (IRCCS), Rome, Italy; ^4^ National Research Council (CNR), Institute of Biochemistry and Cell Biology, Monterotondo, Rome, Italy; ^5^ Consiglio Nazionale delle Ricerche (CNR)-Institute of Cell Biology and Neurobiology, Istituto di Ricerca e Cura a Carattere Scientifico (IRCCS) Fondazione Santa Lucia, Rome, Italy; ^6^ Tumor Immunology Unit , Children Hospital Bambino Gesù, RomaLM, Rome, Italy; ^7^ Department of Life Sciences and Public Health, Catholic University of the Sacred Heart, Rome, Italy

**Keywords:** Che-1, Nectin 1, NK cells, immune response, NK killing activity

## Abstract

**Introduction:**

AATF/Che-1 over-expression in different tumors is well known and its effect on tumorigenicity is mainly due to its central role demonstrated in the oncogenic pathways of solid tumors, where it controls proliferation and viability. The effect exerted by tumors overexpressing Che-1 on the immune response has not yet been investigated.

**Methods:**

Starting from ChIP-sequencing data we confirmed Che-1 enrichment on Nectin-1 promoter. Several co-cultures experiments between NK-cells and tumor cells transduced by lentiviral vectors carrying Che-1-interfering sequence, analyzed by flow-cytometry have allowed a detailed characterization of NK receptors and tumor ligands expression.

**Results:**

Here, we show that Che-1 is able to modulate the expression of Nectin-1 ligand at the transcriptional level, leading to the impairment of killing activity of NK-cells. Nectin-1 down-modulation induces a modification in NK-cell ligands expression able to interact with activating receptors and to stimulate NK-cell function. In addition, NK-cells from Che-1 transgenic mice, confirming a reduced expression of activating receptors, exhibit impaired activation and a preferential immature status.

**Discussion:**

The critical equilibrium between NK-cell ligand expression on tumor cells and the interaction with NK cell receptors is affected by Che-1 over-expression and partially restored by Che-1 interference. The evidence of a new role for Che-1 as regulator of anti-tumor immunity supports the necessity to develop approaches able to target this molecule which shows a dual tumorigenic function as cancer promoter and immune response modulator.

## Introduction

Strategies aimed at affecting the ability of tumor cells to escape from the immune surveillance represent a promising approach in support of current therapies (1–3). Acute lymphoblastic leukemia (ALL) exploits various mechanisms to avoid immune recognition and destruction by the immune system, affecting the phenotypic and functional characteristics of innate and adaptive immune cells (4, 5). A developing leukemia impairs key components of the immune system responsible for anticancer response, particularly in patients poorly responding to treatment or experiencing relapse (6). Among the interactions between leukemia cells and immune system cell populations, the one involving natural killer (NK)-cells is emerging as central in ALL immune-surveillance (7–11). NK-cells are innate lymphoid cells that recognize and kill virus-infected or malignant target cells (12, 13). The NK-cells ability to lyse transformed cells in the absence of antigen-specificity makes them important candidates for treatment of different cancers (14). The ability of NK-cells to kill ALL blasts depends on the balance between the activating and inhibitory receptors on NK-cells, as well as on the presence of their corresponding ligands on ALL cells (8, 15). Many studies have reported down-regulation of activating receptors in peripheral blood NK-cells of patients with hematological malignancies (16–19). NKG2D is an activating immune-receptor expressed on NK-cells able to bind MHC class I-related proteins (MICA and MICB) and ULBP proteins poorly expressed by normal cells, but frequently upregulated in tumor cells (20–22). DNAM-1 receptor has a major costimulatory function exerted through the binding with PVR and Nectin-2 (CD112) ligands on target cells (23–25). ALL blasts escape from NK-cell-mediated killing, predominantly by downregulating the ligands of NK-cell-activating receptors. However, it is of note that also inhibitory receptors act as essential immune check-points (8, 15, 20, 26, 27). Among the NK-cell ligands, Nectins belong to a family of cell-adhesion molecules that can also serve as virus receptors (28, 29). Their expression could represent a potential cancer biomarker, since they are overexpressed on a variety of tumor cells of different origin and can be recognized by activating and inhibitory paired-receptors expressed on NK-cells (30, 31). Tumor cell survival can benefit from modulation of the expression levels of Nectins, thereby influencing subsequent Nectin-mediated signaling, leading to dampened immune response (28, 32). In particular, Nectin-1 (CD111), normally expressed in various epithelial tissues, shows lower expression in tumors of epithelial origin, suggesting a role in reduced cell-cell adhesion, which favors both invasiveness and metastasis (33, 34). In different tumor contexts, such as that of pediatric and adult brain tumors, Nectin-1 was found upregulated (35). A similar modulation was observed also for Nectin-2 (CD112) that, when overexpressed, facilitates tumor cell proliferation, increases invasiveness and migration (36, 37). Thus, the expression of Nectin family proteins can be exploited by tumor cells to evade tumor immune surveillance (28).

Whether an RNA polymerase II binding protein can be involved in immune response is still an unexplored field. AATF/Che-1 (Che-1) has a consolidated role in tumorigenesis of solid tumors and is now clearly involved in the c-Myc-directed oncogenesis in pediatric B-Cell ALL (BCP-ALL) (38). Although ubiquitously expressed, Che-1 overexpression in tumor cells exerts a different contribution in specific oncogenic transcriptional machineries, inducing the expression of cancer genes or upregulating the expression of genes controlling survival functions as cell proliferation (39–42). Che-1-dependent modulation of genes expressing ligands involved in stimulation of the immune system is a field still poorly investigated, although it could offer clues for the identification of new mechanisms of action explaining the meaning of its overexpression in the tumor context. In cancer therapy, it is now evident that targeting pathways of tumorigenesis has limited efficacy, while targeting the cross-talk between tumor and immune cells can strongly improve the current therapies. In this context, we hypothesized that Che-1 could favor tumorigenesis by controlling the expression of membrane-located ligands able to inactivate the anti-tumor immune response. Here, we show that the modulation of Che-1 expression in tumor cells affects the NK-cell-mediated anti-tumor activity by influencing the Nectin-mediated tumor immune surveillance pathways.

## Materials and methods

### Cell lines

LAL-B cell line was obtained by Epstein barr transduction of bone marrow mononuclear cells derived by BCP-ALL patient (Aut. N. 495 11/04/2019). NALM-6 cell line was bought from ATCC (CRL-3273);. NALM-18 cell line was kindly provided by Dr Pende D. (IRCCS San Martino, Genoa, Italy). All cell lines were cultured in RPMI-1640 medium supplemented with 10% FBS (Euroclone, IT), 1% penicillin/streptomycin (Euroclone, IT) and 1% L-glutamine (Euroclone, IT).

All cell lines were tested for mycoplasma contamination by PCR with the following primers:

Forward 5’-ACTCCTACGGGAGGCAGCAGTA-3’

Reverse 5’-TCGACCATCTGTCACTCTGTTAAC-3’

### Antibodies

- Rabbit anti-human AATF/Che-1 antibody (Cat# A301-031A Bethyl, USA)
- Rabbit anti-human Che-1 antibody (43)
- Rabbit anti-P-Erk 1/2 antibody (#9101 Cell Signaling, Euroclone, IT)
- Rabbit anti-Erk 1/2 antibody (#9102 Cell Signaling, Euroclone, IT)
- Rabbit anti-p21 antibody (#2947 Cell Signaling, Euroclone, IT)
- Mouse anti-β-actin antibody (clone AC-15, Sigma – Aldrich, Merck, IT)
- HRP-conjugated anti-Gapdh antibody (MAB-10578, Immunological Sciences, SIC, IT)
- PE-Vio615-conjugated anti-human CD111 antibody (Clone # REA1210, Miltenji Biotech, DE)
- PE-Vio770-conjugated mouse anti-human CD19 antibody (Clone# LT19, Milteniyi Biotec, DE)
- BV421-conjugated mouse anti-human CD19 antibody (Clone# HIB19, BD Biosciences, CA-USA)
- BUV395-conjugated mouse anti-human CD3 antibody (Clone SP34-2, BD Biosciences, CA-USA)
- BV605-conjugated mouse anti-human CD314 (NKG2D) antibody (Clone# 1D11, BD Biosciences, CA-USA)
- FITC-conjugated mouse anti-human CD19 antibody (Clone# CB19, Immunological Science, SIC, IT
- PE-Cy7-conjugated mouse anti-human CD226 (DNAM) antibody (Clone# 11A8, BioLegend, CA-USA)
- APC-conjugated rat anti-human CD96 (TACTILE) antibody (Clone# 3.3, BioLegend, CA-USA)
- PE-Vio615-conjugated anti-human CD111 (Nectin-1) antibody (Clone# REA1210, Milteniyi Biotec, DE)
- APC-conjugated mouse anti-human CD112 (CD112) (Clone# R2.477, Invitrogen, IT)
- AlexaFluor-647-conjugated mouse anti-human CD155 (PVR) antibody (Clone# TX24, BD Biosciences, CA-USA)
- eFluor450-conjugated anti-human CD336 (NKp44) antibody (Clone # 44.189 eBioscience Thermo FIsher Scientific, IT)
- BV510-conjugated mouse anti-human CD337 (NKp30) antibody (Clone# p30-15, BD Biosciences, CA- USA)
- APC-conjugated mouse anti-human CD335 (NKp46) antibody (Clone# 9E2, Milteniyi Biotec, DE)
- APC-conjugated mouse anti-human ULBP4 antibody (Clone# 709116, R&D biosystems, BioTechne, IT)
- PE-conjugated mouse anti-human ULBP2-5-6 antibody (Clone# 165903, R&D biosystems, BioTechne, IT)
- PE-conjugated mouse anti-human ULBP1 antibody (Clone# 170818, R&D biosystems, BioTechne, IT)
- BV421-conjugated mouse anti-CD107a antibody (Clone# H4A3, BD Biosciences, CA- USA)
- PE-conjugated anti-human IFNγ antibody (Clone# REA600, Milteniyi Biotec, DE)
- PE-Vio770-conjugated anti-human TNFα antibody (Clone# cA2, Milteniyi Biotec, DE)
- BUV786-conjugated mouse anti-human CD16 (Clone# 3G8, BD Biosciences, CA-USA)
- APC-conjugated mouse anti-human CD45 antibody (Clone# HI30, Immunological Sciences, SIC, IT)
- FITC-conjugated anti-mouse CD19 (MAB-519F, Immunological Science, SIC, IT)
- BUV395-conjugated anti-mouse CD3 (Clone#17A2, BD Biosciences, CA-USA)
- APC-vio770-conjugated anti-mouse NK1.1 antibody (Clone# PK136, Milteniyi Biotec, DE).
- APC-conjugated anti-mouse CD314 (NKG2D) antibody (Clone# REA1175, Milteniyi Biotec, DE)
- BV711-conjugated rat anti-mouse CD155 (DNAM) antibody (Clone# TX56, BioLegend, CA-USA)
- PE-conjugated hamster anti-mouse CD27 (Clone# LG.3A10, BioLegend, CA-USA)
- PE-cy7-conjugated anti-mouse CD11b (Clone# M1/70, eBioscience, ThermoFIsher scientific, IT)
- Anti-human CD314 (NKG2D) antibody, pure (Clone# BAT221, Miltenji Biotec, DE)
- Anti-human CD226 (DNAM) IgM F5, kindly provided by Dr D. Pende

### Chromatin immunoprecipitation assay

Chromatin immunoprecipitation (ChIP) experiments were performed as previously described by Bruno T. et al., 2006 (44) using anti-AATF/Che-1 antibody (Bethyl, USA). Immunoprecipitations with no specific immunoglobulins (Santa Cruz Biotechnology) were performed as negative controls. For quantitative ChIP analysis (ChIP-qRT), 1 μl of purified DNA was used for amplification on a 7500 Fast Real-Time PCR System (Applied Biosystems) using a SYBER Green 2× qPCR Master Mix (Primerdesign, UK). The following human promoter‐specific primers were employed in RT–PCR amplifications:

Nectin 1 promoter forward 5’ – TGCCGGCGATCCGCAACAATG – 3’

Nectin 1 promoter reverse 5’ – TTAACGCTAACCCCTCCCCTC – 3’

### Che-1 interference

siRNA experiments of Che‐1 expression were performed by transfecting a specific pool of three double–stranded RNA oligonucleotides targeting Che-1 (cat. n. 1299003– HSS120157 HSS120158 and HSS120159) or a control sequence (siControl, cat. n. 12935300), purchased from Thermo Fisher Scientific. Transfections were carried out by nucleofection of NALM‐6 and LAL‐B cells using Amaxa 4D‐Nucleofector X Kit L (Lonza, IT) by following the manufacturer’s instructions.

### Western blotting

Cells were treated as described in Bruno T. et al., 2006 (44). Samples were separated by electrophoresis and transferred onto nitrocellulose membranes. After a blocking step in 5% non-fat-dried milk in 0.1% Tween-PBS, membranes were incubated with primary antibodies overnight at 4°C. After three washes in 0.1% Tween-PBS, membranes were incubated with the appropriate HRP-linked secondary antibodies (Bio-Rad, IT) at room temperature for 45 min, washed with 0.1% Tween-PBS and analyzed by chemi-luminescence (GE Healthcare Life Science, IT). Images were acquired using Alliance Mini HD6 system by UVITEC Ltd, Cambridge, equipped with UVI1D Software (UVITEC, 14–630275). The primary antibodies used were: anti-Che-1 (43), and anti-β-actin (Sigma – Aldrich, Merck, IT).

### RNA isolation and quantitative real-time PCR

Total RNA from NALM‐6 and LAL‐B cells was isolated using EuroGOLD TriFast reagent (Euroclone, IT) according to the manufacturer’s instructions. The first‐strand cDNA was synthesized with random primers and M‐MLV reverse transcriptase (Life Technologies, MA). The cDNA was used for quantitative real‐time PCR (qRT–PCR) experiments carried out in a 7500 Fast Real‐Time PCR System (Applied Biosystems, CA). ΔΔCt values were normalized with those obtained from the amplification of the endogenous β‐actin gene. The following human‐specific primers were employed in RT–PCR amplifications:

Nectin 1 forward 5’- GGATGACAAGGTCCTGGTGG- 3’

Nectin 1 reverse 5’- ACTGCACGTTGAGAGTGAGG- 3’

β - actin forward 5’ - GACAGGATGCAGAAGGAGATTACT - 3’

β - actin reverse 5’ - TGATCCACATCTGCTGGAAGGT - 3’

### Lentiviral transduction

Lentiviral vectors pLV-TH (shControl), pLV-shChe-1 TH (45) were produced as previously described (shChe-1 sequence: nucleotides 824–842). Lentiviral stocks were titrated following standard protocols (45), and, routinely, a viral titer of 10^6^ transducing units per ml (TU/ml) was achieved. Supernatants were collected and employed to infect NALM-6 cells (1x10^6^ cells) in retronectin (Takara Shuzo, JP) pre-coated (7mg/ml) non-tissue culture 24-well plates. Samples were centrifuged at 2000g for 90 minutes. Infection proceeded for 48 hours. Infected cells were harvested and tested for GFP-expression through flow-cytometry analysis.

### Flow-cytometry

Infected NALM-6 and NALM-18 cells lines were collected and analyzed by flow cytometry with PE-Vio615-conjugated anti-CD111 antibody (Miltenji Biotech, DE).

### Human NK-cell isolation

Human NK-cells were isolated from PBMC of healthy donors with the RosetteSep NK-cell enrichment mixture method (Stem-Cell Technologies, IT). NK-cells with purity greater than 90% were stimulated with 100 IU/mL of recombinant human IL2 (PeproTech, FR) for 48 hours at 37°C. NK-cells were maintained in culture with NK MACS medium supplemented with 5% human serum and 1% NK MACS supplement (Miltenyi Biotech, DE).

### NK cells cytotoxicity assay

Cell cytotoxicity assays were performed using as target NALM-6 cell line or K562 cell line and as effector cells NK-cells at different Effector/Target (E/T) cell ratios. Killed cells were evaluated after 4 hours. At the end of the co-culture, the assay was stopped by chilling cells on ice, and Propidium Iodide (PI) was added to each sample immediately before acquisition in order to identify the percentage of target cell lysis, as previously described (Ingegnere T Front Immunol 2019). For each set of experiments, all the acquisitions (5,000 target cells/sample) were performed within 20 min. Experiments aimed to study the involvement of DNAM-1 and NKG2D in NK-cell cytotoxicity against NALM-6 siChe-1 cells were performed after 30 minutes inoculation of NK-cells with F5 anti DNAM-1 or anti- NKG2D (BAT221) antibodies.

### NK-cells co-culture assay

For NK receptors expression detection, NK-cells were plated at 1×10^5^ cells in 96-well plates. NALM-6 cells were added at the indicated ratios. Following 16 hours of incubation at 37°C, NK and NALM-6 cells were collected and assessed by flow-cytometry. BV421 or PE-Vio770-conjugated anti-CD19 with GFP expression were used for target cells exclusion. NK-cells (CD19-/GFP-) were evaluated by BV605-conjugated anti-CD314 (NKG2D), PE-Cy7-conjugated anti-CD226 (DNAM), APC-conjugated anti-CD96 (TACTILE). For Ligands expression detection, NALM-6 cells were plated at 1×10^5^ cells in 96-well plates. NK-cells were added at the indicated ratios. Following 16 hours of incubation at 37°C, NK and NALM-6 cells were collected and assessed by flow-cytometry. BV421 or PE-Vio770-conjugated anti-CD19 with GFP expression were used for target cells selection. NALM-6 siCtrl or siChe-1 (CD19+/GFP+) were evaluated by PE-Vio615-conjugated anti-CD111 (Nectin-1), APC-conjugated anti-CD112 (Nectin-2) and AlexaFluor-647-conjugated anti-CD155 (PVR).

### NK-cells degranulation assay

For degranulation assay NK-cells were plated at 1×10^5^ cells/well in 96-wells plates. NALM-6 cells were added at the indicated ratio and incubated for 3 hours. After one hour the cells were treated with Golgi Stop (BD Biosciences, CA-USA). Thereafter, cells were labeled with PE-Vio770-conjugated anti-CD19, and BV421-conjugated anti-CD107a antibody (BD Biosciences, California, USA) for 20 min at 4°C, followed by flow-cytometric analysis. For intra-cytoplasmatic evaluation of IFNγ and TNFα, cells were fixed and permeabilized with Fix/perm buffer (eBioscience, ThermoFisher scientific, IT) and then labeled with PE-Conjugated anti-IFNγ and PE-Vio770-conjugated anti-TNFα, BUV786-conjugated anti-CD16, APC-conjugated anti-CD45 for 20min at 4°C.

### Transgenic mouse strain generation

All animal studies were approved by the Institutional Animal Care of the Regina Elena National Cancer Institute and by the Government Committee of National Minister of Health and were conducted according with EU Directive 2010/63/EU for animal experiments.

To generate Eμ-Che-1 transgenic mice (C57Bl/6xDBA2 strain) Che-1 was fused to an immunoglobulin enhancer Eμ. After genomic DNA extraction of tail biopsies, the positive founder animals were identified by PCR using the following primers specific for the transgenes:

oligonucleotide up: 5’-CTTCATACCATCCTCTGTGCTTC-3’

oligonucleotide down: 5’-GCTTTTCTAGAGGTGGTTTTGC -3’

Eμ-Che-1 transgenic mice were interbred with MITO-Luc reporter mice (46) to obtain Eμ-Che-1/MITO-Luc (MITO/Che1+/+).

After genomic DNA extraction of tail biopsies, the positive founder animals were identified by PCR using the following primers specific for the transgenes:

oligonucleotide up: 5’-TGTAGACAAGGAAACAACAAA-GCCTGGTGGCC-3’

oligonucleotide down: 5’-GGCGTCTTCCATTTTACCAACAG-TACCGG-3’

MITO/Che+/+ and MITO/Che1+/- used as negative control were subjected to longitudinal *in vivo* imaging sessions at 11 weeks of age.

### *In vivo* imaging

For *in vivo* Bioluminescence imaging (BLI), mice were anesthetized and 75 mg/kg of d-luciferin (Caliper Life Sciences, PerkinElmer, USA) was injected intra-peritoneally. Ten minutes later, quantification of light emission was acquired for 5 min. Signal was detected using the IVIS Lumina II CCD camera system and analyzed with the Living Image2.20 software package (Caliper Life Sciences, PerkinElmer, USA). Photon emission was measured in specific regions of interest (ROIs). Data were expressed as photon/second/cm2/steradiant (p/s/cm2/sr). The intensity of bioluminescence was color coded for imaging purposes; the scale used in each experiment is reported in each figure.

### NK-cells extraction from murine spleen

Murine spleen cells were extracted from MITO/Che1+/+ and MITO/Che1+/- mouse models, and mononuclear cells were obtained from murine spleen cells using FICOLL method. The expression of Murine NK receptors were evaluated through flow-cytometric analysis. We analyzed NK-cells selecting CD3-/CD19- using FITC-conjugated anti-CD19 and BUV395-conjugated anti-CD3. Then from CD3-/CD19- cells we selected NK1.1 positive cells using APC-vio770-Conjugated anti-NK1.1. Murine NK cells were evaluated for NKG2D and DNAM expression using APC-conjugated anti-NKG2D and BV711-conjugated anti-DNAM.

For murine NK activity we selected NK-cells through the same gating strategy used for NK receptor evaluation. NK-cells activity was evaluated using CD27 and CD11b expression using PE-conjugated anti-CD27 and PE-cy7-conjugated anti-CD11b antibodies..

### Statistical analysis

All statistical tests were carried out using GraphPad Prism version 5.0 for Windows, GraphPad Software, San Diego California, USA (www.graphpad.com). Probability values generated by Student’s t‐test considered to be statistically significant are *P ≤ 0.05; **P ≤ 0.01; ***P ≤ 0.001

## Results

### Che-1 transcriptionally controls Nectin-1 expression

**Figure 1:**
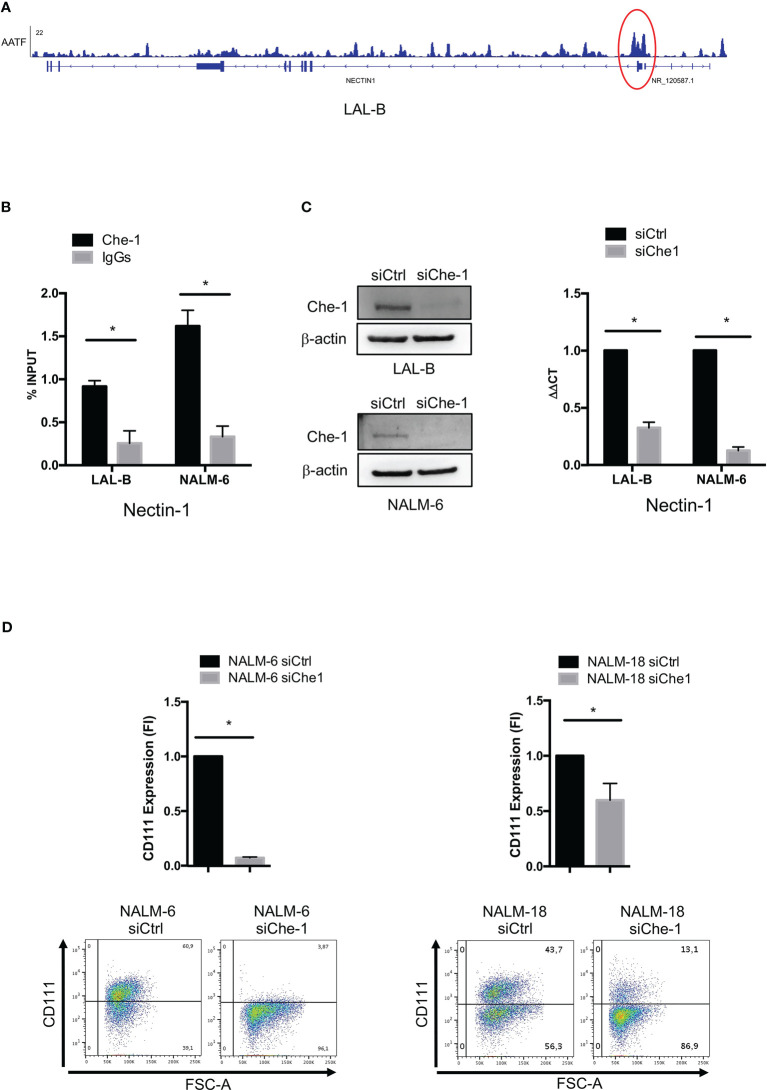
Che-1 transcriptionally controls Nectin-1 (CD111) expression. **(A)** Genome Browser screenshot of ChIP-seq signal on Nectin-1 promoter extracted by ChIP-seq assay previously performed in LAL-B cell line (38). **(B)** ChIP assay performed in LAL-B and NALM-6 cell lines showing Che1 enrichment on Nectin-1 promoter. **(C)** Left: Western Blot for Che-1 expression in LAL-B and NALM-6 cell lines upon Che-1 interference. Right: Real-time-PCR for Nectin-1 sequence in LAL-B and NALM-6 cells interfered with Che-1 expression. **(D)** Nectin-1 (CD111) evaluation by flow cytometry in NALM-6 and NALM-18 cell lines transduced with siChe-1 or siCTRL lentiviral vector (n=3); Graph: flow-cytometry of CD111 expression in one representative plot of CD111 expression out of 3 performed. (*P ≤ 0.05; **P ≤ 0.01; ***P ≤ 0.001).

In order to find evidence of Che-1 involvement in anti-tumor immune response, we analyzed the Chromatin immune-precipitation-sequencing (ChIP-seq) data (38) obtained in the primary BCP-ALL cell line (LAL-B), to identify a possible enrichment of Che-1 on the promoter sequence of genes belonging to immune check-point regulation. Data analysis revealed the presence of Che-1 on Nectin-1 promoter (**Figure 1A**) as confirmed by ChIP-assay performed in LAL-B cell line and in NALM-6, another BCP-ALL cell line (**Figure 1B**). To understand the mechanism of regulation between the two molecules, we down-modulated the expression of Che-1 for 72 hours in the LAL-B and NALM-6 cell lines (**Figure 1C** left panel) and evaluated Nectin-1 gene modulation. We found that Nectin-1 resulted down-regulated upon 72 hours of Che-1 interference (**Figure 1C** right panel). In NALM-6 cell line, by lentiviral transduction, we inhibited Che-1 expression (NALM-6 siChe-1)as shown in **Supplementary Figure 1A**, and analyzed the surface expression of Nectin-1 protein by flow-cytometry analysis. We show that Che-1 interference resulted in Nectin-1 down-modulation, when compared with the controls in which cells were transduced with non-target lentiviral vector (NALM-6 siCtrl). The same result was obtained in NALM-18 BCP-ALL cell line (**Figure 1D**). These data confirm that Che-1 sustains its tumorigenic function also by controlling the immune check-point ligands expression on blast cell membrane.

### Che-1 overexpression impairs NK-cell killing activity

**Figure 2:**
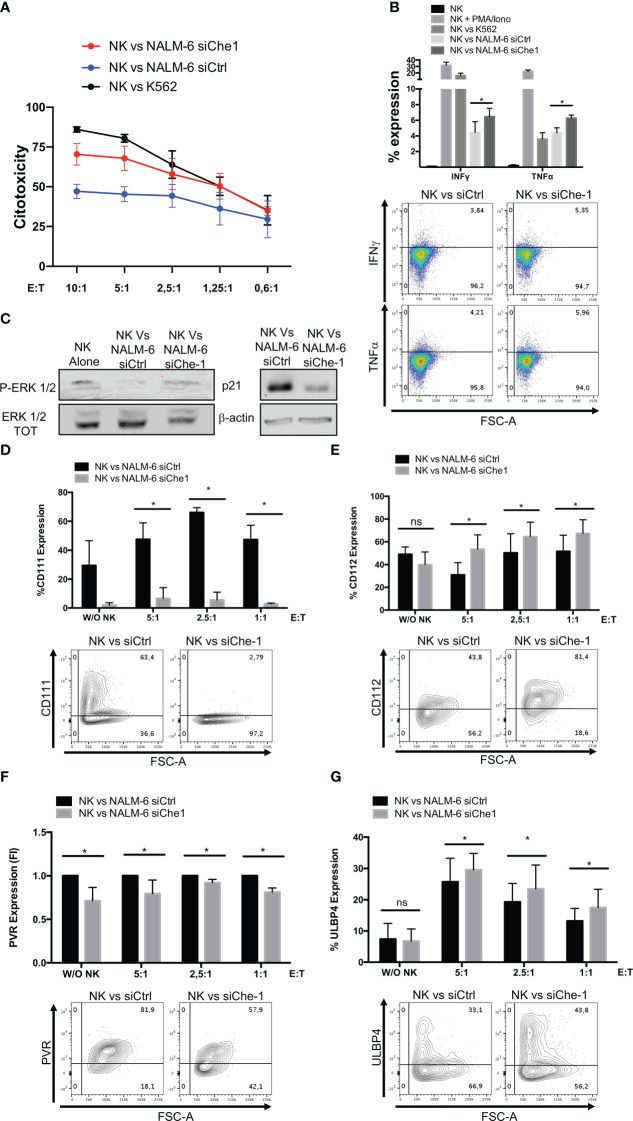
Che-1-dependent Nectin-1 down-modulation modifies NK-cell ligand expression. **(A)** Citotoxicity assay of NK-cells (CD19-/GFP-) after a 4-hours-culture with NALM-6 siCtrl or siChe-1 at different Effector : Target (E:T) ratio (10:1, 5:1, 2,5:1, 1,25:1, 0,6:1) (n=3). K562 cell line used as positive control condition **(B)** Graph: flow-cytometry of intra-cytoplasmatic IFNγ and TNFα % of expression in NK-cells co-cultured for 4 hours with NALM-6 siCtrl or siChe-1 E:T 1:1 (n=3). NK-cells alone, stimulated with PMA/Ionomycin (PMA 25ng/ml; Ionomycin 1μg/ml) and co-cultured with K562 were used as control conditions. One representative plot of IFNγ and TNFα expression out of 3 performed. **(C)** One representative p-Erk 1/2 and p21 WB of 3 performed in NK-cells sorted upon 24 hours of co-culture with NALM-6 siCtrl and siChe-1. NK-cell alone sample was used as p-Erk 1/2 basal level. Anti-Erk 1/2 Total (TOT) and anti Actin antibodies were used as loading control. **(D)** Graph: flow cytometry of CD111 (CD19+/GFP+) after a 16-hour co-culture with NK-cells at different E:T ratio (5:1, 2,5:1, 1:1) (n=3). One representative plot of CD111 expression out of 3 performed. Basal CD111 expression was measured in w/o NK cell condition **(E)** Graph: CD112 evaluation by flow-cytometry of NALM-6 siCtrl and siChe-1 (CD19+/GFP+) after a 16-hour co-culture with NK-cells at different E:T ratio (5:1, 2,5:1, 1:1) (n=3). One representative plot of CD112 expression out of 3 performed. Basal CD112 expression was measured in w/o NK cell condition **(F)** Graph: flow-cytometry of NALM-6 siCtrl and siChe-1 (CD19+/GFP+) after a 16-hour co-culture with NK-cells at different E:T ratio (5:1, 2,5:1, 1:1) (n=3). One representative plot of PVR expression out of 3 performed. Basal PVR expression was measured in w/o NK cell condition **(G)** Graph: flow-cytometry of ULBP4 of NALM-6 siCtrl and siChe-1 (CD19+/GFP+) after a 16-hour co-culture with NK-cells at different E:T ratio (5:1, 2,5:1, 1:1) (n=3). One representative plot of ULBP4 expression out of 3 performed. Basal ULBP4 expression was measured in w/o NK cell condition. (*P ≤ 0.05; **P ≤ 0.01; ***P ≤ 0.001; ns, not significant).

Since Nectin-1 appears to play an increasing role in tumor immune response (47), we studied its mechanism of action by analyzing NK-cell function. We performed co-culture experiments of NK-cells obtained from peripheral blood of healthy donors with NALM-6 siChe-1 cells or NALM-6 siCtrl cells, as negative control. Cytotoxicity assay demonstrated that NK-cells showed a reduced killing activity when in co-culture with Che-1-overexpressing NALM-6 cell line that is rescued when in co-culture with Che-1-depleted cell line (**Figure 2A**). Degranulation assay, performed by evaluating CD107a expression on NK-cells, confirmed that Che-1 silenced cells resulted more susceptible to NK-cell degranulation activity when compared with the control condition. Of note, this occurred also at 5:1 and 2,5:1 Effector: Target (E:T) ratio in which NK-cells are quantitatively favored (**Supplementary Figure 2A**). We further investigated whether this phenomenon reflected an increased capability of NK-cells of releasing effector molecules (IFNγ and TNFα) under the same experimental conditions. Flow-cytometry analysis revealed that the intracellular amount of these two cytokines was significantly increased after co-culture with Che-1 down-regulated cells as compared to the control one (**Figure 2B**). To deeper understand the effect exerted by Che-1 on NK-cell function, we measured NK proliferation by p-Erk1/2 expression. After a 24-hour co-culture with NALM-6-siCtrl, NK-cell proliferation was strongly reduced if compared with siChe-1 condition where p-Erk1/2 comes-back to the level expressed by NK-cell cultured alone. In addition p21, used as marker of cell cycle arrest, showed high expression level in NK-cells co-cultured with NALM-s siCtrl if compared with siChe condition, confirming the control exerted by Che-1 overexpressing cells on NK-cells proliferation (**Figure 2C**). In order to better understand the mechanism of action responsible of this functional effect, we performed longer co-culture experiments (16 hours), to study the expression of ligands either in the presence or in the absence of Che-1.

As shown in **Figure 2D**, in siChe-1 experimental condition, we confirmed that Nectin-1 expression was reduced after 16h of co-culture. Conversely, Nectin-2 expression was increased (**Figure 2E**), suggesting a possible mechanism of compensation in the blast cells. This is supported by the known trans-interaction mechanism occurring among the Nectin family members (48). Our hypothesis was that up-regulation of Nectin-2 could result in binding of DNAM-1 receptor on NK-cells leading to their activation. This hypothesis is also supported by the lower expression of PVR on siChe-1 cells (**Figure 2F**). A second interesting effect was observed in the modulation of ULBP molecules on siChe-1 cells. In particular, among the members of ULBP family, we observed in NALM-6 siChe-1 cells an increase of ULBP4 expression (**Figure 2G**) (49), while ULBP1, 2, 5, 6 were down-modulated (**Supplementary Figures 2B, C**). Therefore, data on ligand modulation revealed that Che-1 can re-modulate ligand expression on blast cell membrane through the transcriptional inhibition of Nectin-1.

### NKG2D and DNAM-1 receptors are involved in Che-1-driven NK-cells inhibition

**Figure 3:**
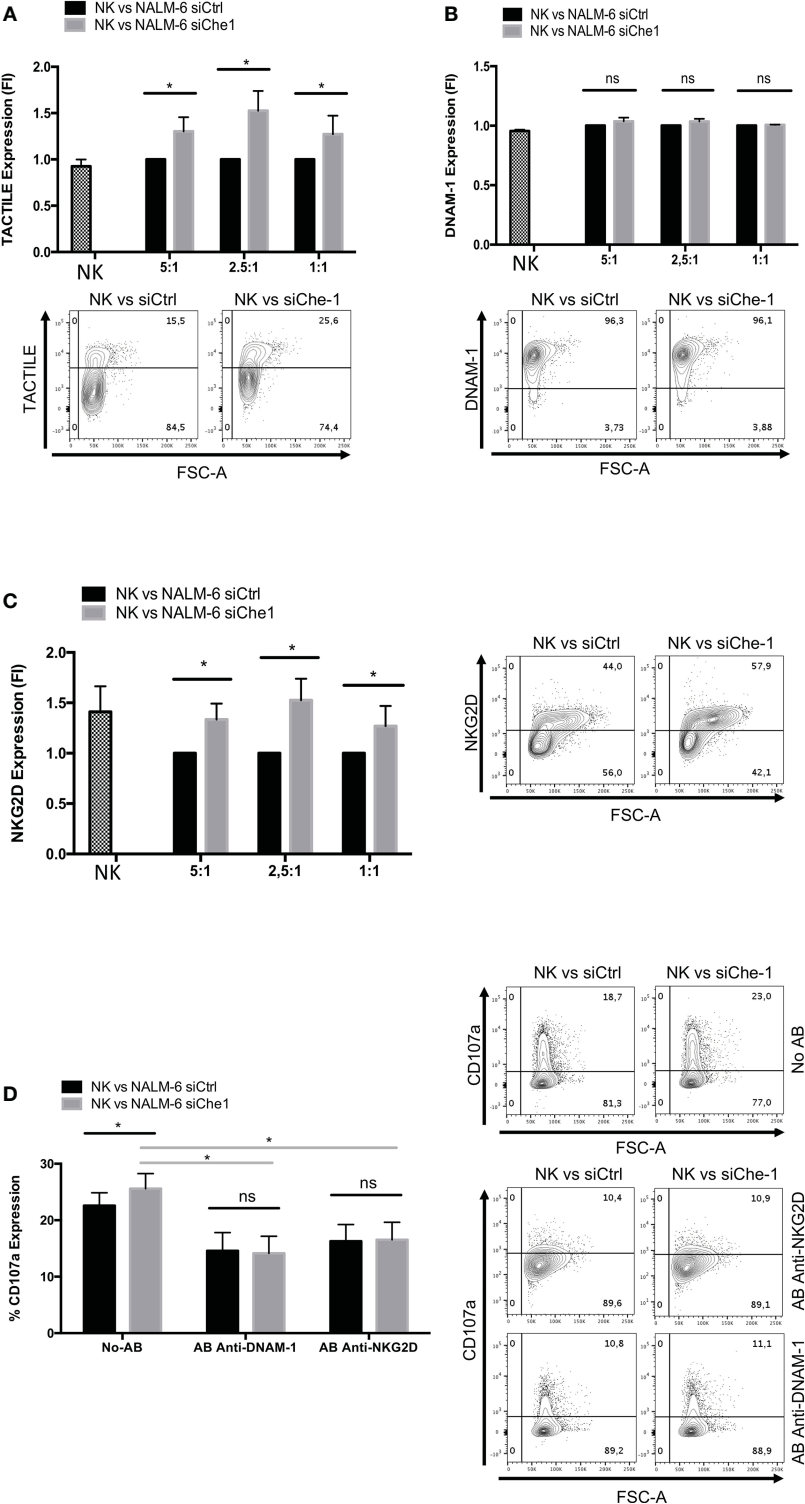
NKG2D and DNAM-1 NK receptors are induced when in co-culture with Che-1 interfered cells. **(A)** Graph: flow-cytometry of TACTILE expression, measured as Fold of Induction (FI), of NK-cells (CD19-/GFP-) after a 16-hour co-culture with NALM-6 siCtrl and siChe-1 NK-cells at different E:T ratio (5:1, 2,5:1, 1:1) (n=3). One representative plot of TACTILE expression out of 3 performed. Basal TACTILE expression was measured in NK alone condition **(B)** Graph: of DNAM-1 expression, measured as FI of in flow-cytometry NK-cells (CD19-/GFP) after a 16-hour co-culture with NALM-6 siCtrl and siChe-1 NK-cells at different E:T ratio (5:1, 2,5:1, 1:1) (n=3). One representative plot of DNAM-1 expression out of 3 performed. Basal DNAM-1 expression was measured in NK alone condition **(C)** Graph: NKG2D expression by flow-cytometry measured as FI in NK-cells (CD19-/GFP-) after 16-hour of co-culture with NALM-6 siCtrl and siChe-1 NK-cells at different E:T ratio (5:1, 2,5:1, 1:1) (n=3). One representative plot of NKG2D expression out of 3 performed. Basal NKG2D expression was measured in NK alone condition **(D)** Graph: CD107a evaluation by flow-cytometry of NK-cells (CD19-/GFP-) after a 4 hours of co-culture with NALM-6 siCtrl or siChe-1 at 1:1 E:T ratio in presence of anti-NKG2D or anti-DNAM-1 masking antibodies, respectively, to block interaction with their ligands. (n=3). One Representative plot of CD107a expression of 3 performed. (*P ≤ 0.05; **P ≤ 0.01; ***P ≤ 0.001; ns, not significant).

The modulation of ligand expression due to Che-1 interference prompted us to investigate also the possible effect exerted on NK-cell receptor expression and function. Starting from the paired Nectin-1 receptor, TACTILE, we observed an increase in the level of expression of TACTILE on NK-cells co-cultured with siChe-1 cells probably due to siChe-1-dependent reduced expression of its preferred ligand, Nectin-1 (**Figure 3A**).

In addition, based on the previous result (**Figure 2E**) in which PVR expression was impaired in NK-cells co-cultured with siChe-1 NALM-6, we also assessed DNAM-1 expression on NK-cells. Notably, DNAM-1 expression was not modified by Che-1 expression modulation (**Figure 3B**). This result could be due to a strong up-regulation of Nectin-2 (**Figure 2E**) occurring upon siChe-1-mediated Nectin-1 inhibition. Similarly to TACTILE, also NKG2D expression was increased on NK-cells upon 16 hours of co-culture with NALM-6 siChe-1 as compared to control cells (**Figure 3C**). The others NK receptors belonging to the NCR family (NKp30, NKp44 and NKp46) are not affected as shown in **Supplementary Figures 3A–C**.

To further understand whether these two pathways could be responsible of NK-cell re-activation after Che-1 depletion, we evaluated the NK-cell cytolytic activity under the same previous experimental conditions, either in the presence or in the absence of NKG2D and DNAM-1 monoclonal antibodies (mAbs). These masking mAbs are able to block the interactions between NK activating receptors and their ligands (20, 50). As shown in **Figure 3D**, mAb-mediated masking of DNAM-1 or NKG2D inhibited NK-cell degranulation against siChe-1 NALM-6 cell line. These results suggest that Che-1 exerts its inhibitory function on the immune response by affecting the two principal pathways sustaining NK-cell cytolytic activity.

### Che-1-dependent NKG2D and DNAM-1 down-modulation *in vivo*

**Figure 4:**
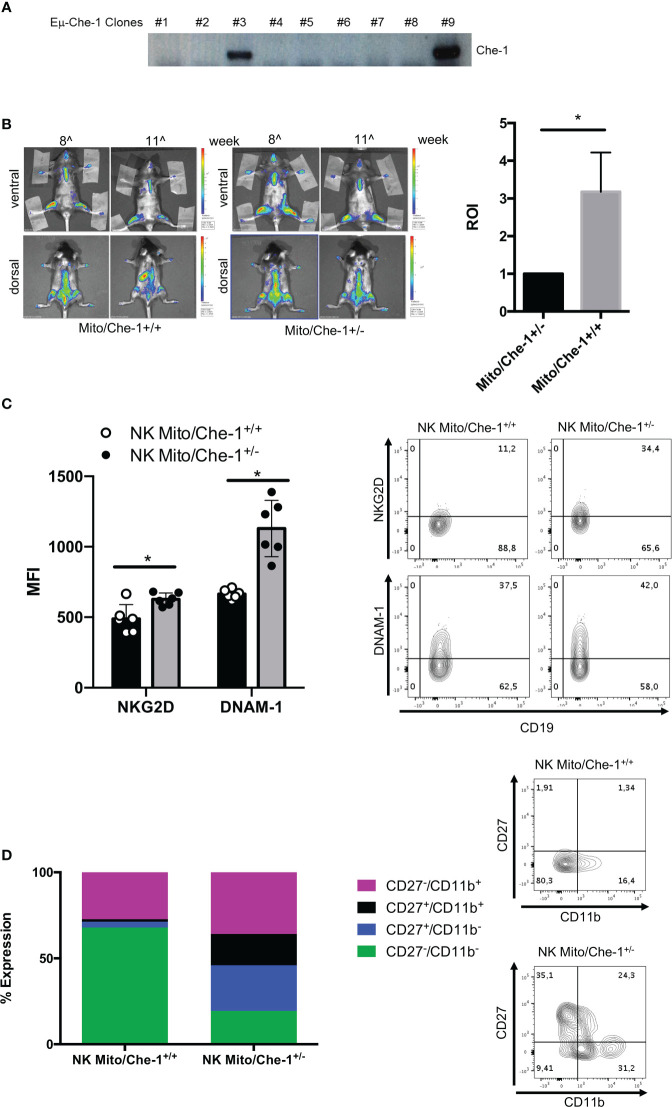
*In vivo* evaluation of Che-1-dependent NK inactivation. **(A)** PCR showing genotyping of Eμ-Che-1 transgenic mice. **(B)** Left: Bio-imaging of MITO/Che-1^+/+^ and MITO/Che-1^+/-^ mice at 11 weeks of age (n=6). Right: graph quantifies spleen luminescence at week 11 (n=6). **(C)**. % of expression of NKG2D and DNAM-1 in NK-cells extracted from spleens of MITO/Che-1^+/+^ and MITO/Che-1^+/-^ mice at week 11 by flow-cytometry (n=3). One representative plot of both receptors out of 3 performed. **(D)** CD27/CD11b evaluation in spleen-derived NK cells: CD27^-^/CD11b^+^ used to detect cytolytic compartment; CD27^+^/CD11b^-^ and CD27^+^/CD11b^+^ for the cytokine production compartment; CD27^-^/CD11b^+^ to measure the maturation status (n=3). One representative plot of the 3 subgroups out of three performed. *P ≤ 0.05; **P ≤ 0.01; ***P ≤ 0.001.

In order to investigate the physiological effect of Che-1 over-expression, we generated a transgenic model where Che-1 was fused with an immunoglobulin enhancer (Eμ), to select the B-cell compartment. **Figure 4A** shows that Che-1 is expressed in two out of nine clones. Taking advantage of the MITO-luc reporter mouse model, previously generated in our lab (46), we crossed them with the EμChe-1 transgenic model with the aim to obtain mice over-expressing Che-1 in the lymphoid organs using a system that allows to monitor cellular proliferation. As expected, MITO/Che-1^+/+^ mice showed high proliferation rate monitored as spleen luminescence demonstrating that Che-1 is strongly involved in B-cell proliferation even in a non-tumoral context. Conversely, MITO/Che-1^+/-^ mice, not carrying Che-1 overexpression, showed a sharply reduced proliferation rate and were used as negative control (**Figure 4B**). In addition, we evaluated the relation between Che-1 over-expression and NK-cells in this *in vivo* setting where the MITO-Luc system allows to monitor the hyper-proliferative status due to Che-1 overexpression. **Figure 4C** shows that spleen-derived NK-cells from MITO/Che-1^+/-^ display higher NKG2D and DNAM-1 expression as compared with MITO/Che-1^+/+^ mice, thus confirming the *in vitro* data using human NK-cells. Furthermore, assessment of the murine NK-cell activation status through the analysis of CD27/CD11b expression (51) revealed that in the MITO/Che-1^+/+^ mice the NK-cells were poorly activated as compared to MITO/Che-1^+/-^ mice (**Figure 4D**). Indeed, both CD27^-^/CD11b^+^ cytolytic NK-cells and CD27^+^ CD11b^+^ or CD11b^-^ NK-cells (mainly releasing cytokines) were reduced in MITO/Che-1^+/+^ mice. These data confirm the *in vitro* data, showing a reduced NK-cell activation when co-cultured with Che-1 overexpressing cells. In conclusion, the population of immature NK-cells identified by CD27^-^/CD11b^-^ is higher in MITO/Che-1^+/+^ mice than in MITO/Che-1^+/-^ mice, suggesting that Che-1 overexpression exerts a control on NK-cell development and function.

## Discussion

There is increasing evidence regarding the Che-1 over-expression in tumors and its pivotal role in the transcriptional machinery to cooperate in tumorigenic pathways (39, 41). Che-1 characterization in hematological tumors of adults like multiple myeloma, and of pediatric ones such as BCP-ALL was recently defined. In a previous work (38) we demonstrated that Che-1 over-expression is a crucial inducer of blast cell proliferation. We showed that Che-1 is a member of the c-Myc controlled oncogenic pathway and its down-regulation can interfere with c-Myc-dependent regulation of BCP-ALL tumorigenesis. Despite the numerous experimental evidences of the tumorigenic role of Che-1, the effect of its over-expression on the tumor microenvironment has not been investigated. Data obtained by ChIP-seq experiments in a primary BCP-ALL cell line captured our attention showing Che-1 connection with molecules involved in immune response. The discovery of Che-1 enrichment on Nectin-1 promoter suggested to further investigate its mechanism of action. Since NK-cells represent a first line of defenses against tumor growth and metastasis, it is important to study mechanisms which may interfere with anti-tumor immune responses to allow the development of new immunotherapeutic strategies able to rescue anti-tumor function.

This study demonstrates a new mechanism through which tumor cells may increase their ability to escape immune surveillance by modifying the interactions between ligands on tumor cells and the corresponding receptors on NK-cells. The role of Nectin-1 in the tumor context is still poorly investigated; however, we demonstrated that silencing of Che-1 on tumor cells resulted in down-regulation of Nectin-1, while inducing Nectin-2 overexpression as a result of the heterophilic trans-interaction occurring among Nectin family members. We speculate that this effect may be the starting point of a recalibrated ligand expression pattern able to modulate activating NK receptors and, as a consequence, NK-cell anti-tumor activity. The recruitment of NK-cells is attractive in cancer treatment and a key function of NK-cell therapy is widely appreciated as the therapeutic targeting of NK-cell ligands. In addition, regarding the paucity in healthy tissues, ligands for activating NK receptors may represent valid target antigens on malignant cells for antibody-based approaches. The blockade of the interactions between NKG2D and its ligands could lead to reduced anti-tumor response. In support of these results, our *in vivo* experiments confirm a reduced expression of activating receptors on NK-cells from Che-1 transgenic mice. These cells exhibit an impaired activation and a preferential immature status.

Our study demonstrates that Che-1 is upstream of the mechanism orchestrating the re-modulation of NK-ligand expression, thus proposing Che-1 as an efficient bi-specific target able to affect tumor cell viability and, at the same time, to favor NK-mediated immune responses. The difficulty encountered in the last years to develop an approach able to target Che-1 in view of its nuclear localization could now be overcome by the delivery of CRISPR/Cas-9 RNP complex to down-regulate its expression (52). Delivery through gold-nanoparticles is able to guarantee tumor cell entrance in solid and hematological cancers allowing the validation of the system’s efficacy in pre-clinical murine tumor models.

## Data availability statement

The original contributions presented in the study are included in the article/**Supplementary Material**, further inquiries can be directed to the corresponding author/s.

## Ethics statement

The animal study was reviewed and approved by the Institutional Animal Care of the Regina Elena National Cancer Institute and by the Government Committee of National Minister of Health and were conducted according with EU Directive 2010/63/EU for animal experiments.

## Author contributions

MC and NT conceived and performed the experiments, analyzed the data and contributed to the drafting of the manuscript. CS, SI, IM, GS and SG conceived and performed the experiments and analyzed the data. GP, EM, MF and LM contributed to the study design and provided intellectual input; VF and PV conceived the study, designed the experiments and drafted the manuscript. FL provided intellectual input, analyzed the data, supervised study conduction and critically revised the manuscript. All authors contributed to the article and approved the submitted version.

## Supplementary material

Supplementary Figure 1 Che-1 depletion by lentiviral transduction. **(A)** WB analysis of NALM-6 and NALM-18 cell lines transduced with siCtrl or siChe-1 sequences-carrying lentiviral plasmids to inhibit Che-1 expression. Gapdh antibody was used as loading control.

Supplementary Figure 2 siChe-1-dependent CD107a and ULBP family members modulation. **(A)** CD107a expression of NK-cells (CD19-/GFP-) by flow cytometry after a 4-hours-culture with NALM-6 siCtrl or siChe-1 at different Effector : Target (E:T) ratio (n=3). One Representative plot of CD107a expression of 3 performe. NK alone, Nk-cells stimulated with PMA/Ionomycyn and co-cultured were used as control cinditions (Left) (**B** Graph: flow-cytometry of ULBP1 expression of NALM-6 siCtrl and siChe-1 (CD19+/GFP+) after 16 hours of co-culture with NK-cells at different E:T ratio (n=3). One representative plot of ULBP1 expression out of 3 performed. Basal ULBP1 expression was measured in w/o NK cell condition **(C)** Graph: flow-cytometry of ULBP2-5-6 expression of NALM-6 siCtrl and siChe-1 (CD19+/GFP+) after 16-hour co-culture with NK-cells at different E:T ratio (n=3). One representative plot of ULBP2-5-6 expression out of 3 performed. Basal ULBP2-5-6 expression was measured in w/o NK cell condition.

Supplementary Figure 3 Natural Citotoxicity Receptors (NCR) expression. **(A)** NKp30, **(B)** NKp44 and **(C)** NKp46 evaluation by flow cytometry of NK-cells(CD19-/GFP-) after a 16-hour co-culture with NALM-6 siCtrl and siChe-1 at different E:T ratio.

## References

[1] Beatty GL Gladney WL. Immune escape mechanisms as a guide for cancer immunotherapy. Clin Cancer Res (2015) 21:687–92. doi: 10.1158/1078-0432.CCR-14-1860 PMC4334715 25501578

[2] Vinay DS Ryan EP Pawelec G Talib WH Stagg J Elkord E. Immune evasion in cancer: mechanistic basis and therapeutic strategies. Semin Cancer Biol (2015) 35(Suppl):S185–98. doi: 10.1016/j.semcancer.2015.03.004 25818339

[3] Disis ML. Immune regulation of cancer. J Clin Oncol (2010) 28:4531–8. doi: 10.1200/JCO.2009.27.2146 PMC3041789 20516428

[4] Pastorczak A Domka K Fidyt K Poprzeczko M Firczuk M. Mechanisms of immune evasion in acute lymphoblastic leukemia. Cancers (Basel) (2021) 13:1536. doi: 10.3390/cancers13071536 33810515 PMC8037152

[5] Hunter R Imbach KJ Zhou C Dougan J Hamilton JAG Chen KZ. B-cell acute lymphoblastic leukemia promotes an immune suppressive microenvironment that can be overcome by IL-12. Sci Rep (2022) 12:11870–022. doi: 10.1038/s41598-022-16152-z PMC9279427 35831470

[6] Kang SH Hwang HJ Yoo JW Kim H Choi ES Hwang S. Expression of immune checkpoint receptors on T-cells and their ligands on leukemia blasts in childhood acute leukemia. Anticancer Res (2019) 39:5531–9. doi: 10.21873/anticanres.13746 31570447

[7] Malhotra A Shanker A. NK cells: immune cross-talk and therapeutic implications. Immunotherapy (2011) 3:1143–66. doi: 10.2217/imt.11.102 PMC3230271 21995569

[8] Pende D Marcenaro S Falco M Martini S Bernardo ME Montagna D. Anti-leukemia activity of alloreactive NK cells in KIR ligand-mismatched haploidentical HSCT for pediatric patients: evaluation of the functional role of activating KIR and redefinition of inhibitory KIR specificity. Blood (2009) 113:3119–29. doi: 10.1182/blood-2008-06-164103 18945967

[9] Carlsten M Jaras M. Natural killer cells in myeloid malignancies: immune surveillance, NK cell dysfunction, and pharmacological opportunities to bolster the endogenous NK cells. Front Immunol (2019) 10:2357. doi: 10.3389/fimmu.2019.02357 31681270 PMC6797594

[10] Sportoletti P De Falco F Del Papa B Baldoni S Guarente V Marra A. NK cells in chronic lymphocytic leukemia and their therapeutic implications. Int J Mol Sci (2021) 22:6665. doi: 10.3390/ijms22136665 34206399 PMC8268440

[11] Paul S Lal G. The molecular mechanism of natural killer cells function and its importance in cancer immunotherapy. Front Immunol (2017) 8:1124. doi: 10.3389/fimmu.2017.01124 28955340 PMC5601256

[12] Moretta L Montaldo E Vacca P Del Zotto G Moretta F Merli P. Human natural killer cells: origin, receptors, function, and clinical applications. Int Arch Allergy Immunol (2014) 164:253–64. doi: 10.1159/000365632 25323661

[13] Sivori S Pende D Quatrini L Pietra G Della Chiesa M Vacca P. NK cells and ILCs in tumor immunotherapy. Mol Aspects Med (2021) 80:100870. doi: 10.1016/j.mam.2020.100870 32800530

[14] Valipour B Velaei K Abedelahi A Karimipour M Darabi M Charoudeh HN. NK cells: an attractive candidate for cancer therapy. J Cell Physiol (2019) 234:19352–65. doi: 10.1002/jcp.28657 30993712

[15] Sivori S Vacca P Del Zotto G Munari E Mingari MC Moretta L. Human NK cells: surface receptors, inhibitory checkpoints, and translational applications. Cell Mol Immunol (2019) 16:430–41. doi: 10.1038/s41423-019-0206-4 PMC6474200 30778167

[16] Viel S Charrier E Marcais A Rouzaire P Bienvenu J Karlin L. Monitoring NK cell activity in patients with hematological malignancies. Oncoimmunology (2013) 2:e26011. doi: 10.4161/onci.26011 24327939 PMC3850490

[17] Valenzuela-Vazquez L Nunez-Enriquez JC Sanchez-Herrera J Medina-Sanson A Perez-Saldivar ML Jimenez-Hernandez E. NK cells with decreased expression of multiple activating receptors is a dominant phenotype in pediatric patients with acute lymphoblastic leukemia. Front Oncol (2022) 12:1023510. doi: 10.3389/fonc.2022.1023510 36419901 PMC9677112

[18] Lee LJ Hassan N Idris SZ Subbiah SK Seow HF Mohtaruddin N. Differential regulation of NK cell receptors in acute lymphoblastic leukemia. J Immunol Res (2022) 2022:7972039. doi: 10.1155/2022/7972039 35652109 PMC9150999

[19] Gismondi A Stabile H Nisti P Santoni A. Effector functions of natural killer cell subsets in the control of hematological malignancies. Front Immunol (2015) 6:567. doi: 10.3389/fimmu.2015.00567 26594216 PMC4633523

[20] Pende D Spaggiari GM Marcenaro S Martini S Rivera P Capobianco A. Analysis of the receptor-ligand interactions in the natural killer-mediated lysis of freshly isolated myeloid or lymphoblastic leukemias: evidence for the involvement of the poliovirus receptor (CD155) and nectin-2 (CD112). Blood (2005) 105:2066–73. doi: 10.1182/blood-2004-09-3548 15536144

[21] Fan J Shi J Zhang Y Liu J An C Zhu H. NKG2D discriminates diverse ligands through selectively mechano-regulated ligand conformational changes. EMBO J (2022) 41:e107739. doi: 10.15252/embj.2021107739 34913508 PMC8762575

[22] Cho H Chung J Kim S Braunschweig T Kang TH Kim J. MICA/B and ULBP1 NKG2D ligands are independent predictors of good prognosis in cervical cancer. BMC Cancer (2014) 14:957–2407. doi: 10.1186/1471-2407-14-957 25510288 PMC4301905

[23] Shibuya A Campbell D Hannum C Yssel H Franz-Bacon K McClanahan T. DNAM-1, a novel adhesion molecule involved in the cytolytic function of T lymphocytes. Immunity (1996) 4:573–81. doi: 10.1016/s1074-7613(00)70060-4 8673704

[24] Pende D Bottino C Castriconi R Cantoni C Marcenaro S Rivera P. PVR (CD155) and nectin-2 (CD112) as ligands of the human DNAM-1 (CD226) activating receptor: involvement in tumor cell lysis. Mol Immunol (2005) 42:463–9. doi: 10.1016/j.molimm.2004.07.028 15607800

[25] de Andrade LF Smyth MJ Martinet L. DNAM-1 control of natural killer cells functions through nectin and nectin-like proteins. Immunol Cell Biol (2014) 92:237–44. doi: 10.1038/icb.2013.95 24343663

[26] Velardi A Ruggeri L Alessandro Moretta Moretta L. NK cells: a lesson from mismatched hematopoietic transplantation. Trends Immunol (2002) 23:438–44. doi: 10.1016/s1471-4906(02)02284-6 12200065

[27] Sivori S Della Chiesa M Carlomagno S Quatrini L Munari E Vacca P. Inhibitory receptors and checkpoints in human NK cells, implications for the immunotherapy of cancer. Front Immunol (2020) 11:2156. doi: 10.3389/fimmu.2020.02156 33013909 PMC7494755

[28] Duraivelan K Samanta D. Emerging roles of the nectin family of cell adhesion molecules in tumour-associated pathways. Biochim Biophys Acta Rev Cancer (2021) 1876:188589. doi: 10.1016/j.bbcan.2021.188589 34237351

[29] Cifaldi L Doria M Cotugno N Zicari S Cancrini C Palma P. DNAM-1 activating receptor and its ligands: how do viruses affect the NK cell-mediated immune surveillance during the various phases of infection? Int J Mol Sci (2019) 20:3715. doi: 10.3390/ijms20153715 31366013 PMC6695959

[30] Sanchez-Correa B Valhondo I Hassouneh F Lopez-Sejas N Pera A Bergua JM. DNAM-1 and the TIGIT/PVRIG/TACTILE axis: novel immune checkpoints for natural killer cell-based cancer immunotherapy. Cancers (Basel) (2019) 11:877. doi: 10.3390/cancers11060877 31234588 PMC6628015

[31] Pasero C Gravis G Granjeaud S Guerin M Thomassin-Piana J Rocchi P. Highly effective NK cells are associated with good prognosis in patients with metastatic prostate cancer. Oncotarget (2015) 6:14360–73. doi: 10.18632/oncotarget.3965 PMC4546472 25961317

[32] Lozano E Mena M Diaz T Martin-Antonio B Leon S Rodriguez-Lobato L. Nectin-2 expression on malignant plasma cells is associated with better response to TIGIT blockade in multiple myeloma. Clin Cancer Res (2020) 26:4688–98. doi: 10.1158/1078-0432.CCR-19-3673 32513837

[33] Guzman G Oh S Shukla D Valyi-Nagy T. Nectin-1 expression in the normal and neoplastic human uterine cervix. Arch Pathol Lab Med (2006) 130:1193–5. doi: 10.5858/2006-130-1193-NEITNA 16879022

[34] Matsushima H Utani A Endo H Matsuura H Kakuta M Nakamura Y. The expression of nectin-1alpha in normal human skin and various skin tumours. Br J Dermatol (2003) 148:755–62. doi: 10.1046/j.1365-2133.2003.05225.x 12752135

[35] Friedman GK Bernstock JD Chen D Nan L Moore BP Kelly VM. Enhanced sensitivity of patient-derived pediatric high-grade brain tumor xenografts to oncolytic HSV-1 virotherapy correlates with nectin-1 expression. Sci Rep (2018) 8:13930–018. doi: 10.1038/s41598-018-32353-x PMC6141470 30224769

[36] Oshima T Sato S Kato J Ito Y Watanabe T Tsuji I. Nectin-2 is a potential target for antibody therapy of breast and ovarian cancers. Mol Cancer (2013) 12:60–4598. doi: 10.1186/1476-4598-12-60 23758976 PMC3698035

[37] Li M Qiao D Pu J Wang W Zhu W Liu H. Elevated nectin-2 expression is involved in esophageal squamous cell carcinoma by promoting cell migration and invasion. Oncol Lett (2018) 15:4731–6. doi: 10.3892/ol.2018.7953 PMC5840744 29552112

[38] Folgiero V Sorino C Pallocca M De Nicola F Goeman F Bertaina V. Che-1 is targeted by c-myc to sustain proliferation in pre-b-cell acute lymphoblastic leukemia. EMBO Rep (2018) 19:e44871. doi: 10.15252/embr.201744871 29367285 PMC5835840

[39] Bruno T Desantis A Bossi G Di Agostino S Sorino C De Nicola F. Che-1 promotes tumor cell survival by sustaining mutant p53 transcription and inhibiting DNA damage response activation. Cancer Cell (2010) 18:122–34. doi: 10.1016/j.ccr.2010.05.027 20708154

[40] Desantis A Bruno T Catena V De Nicola F Goeman F Iezzi S. Che-1-induced inhibition of mTOR pathway enables stress-induced autophagy. EMBO J (2015) 34:1214–30. doi: 10.15252/embj.201489920 PMC4426481 25770584

[41] Bruno T Valerio M Casadei L De Nicola F Goeman F Pallocca M. Che-1 sustains hypoxic response of colorectal cancer cells by affecting hif-1alpha stabilization. J Exp Clin Cancer Res (2017) 36:32–017. doi: 10.1186/s13046-017-0497-1 28214471 PMC5316229

[42] Passananti C Floridi A Fanciulli M. Che-1/AATF, a multivalent adaptor connecting transcriptional regulation, checkpoint control, and apoptosis. Biochem Cell Biol (2007) 85:477–83. doi: 10.1139/O07-062 17713582

[43] Fanciulli M Bruno T Di Padova M De Angelis R Iezzi S Iacobini C. Identification of a novel partner of RNA polymerase II subunit 11, che-1, which interacts with and affects the growth suppression function of Rb. FASEB J (2000) 14:904–12. doi: 10.1096/fasebj.14.7.904 10783144

[44] Bruno T De Nicola F Iezzi S Lecis D D'Angelo C Di Padova M. Che-1 phosphorylation by ATM/ATR and Chk2 kinases activates p53 transcription and the G2/M checkpoint. Cancer Cell (2006) 10:473–86. doi: 10.1016/j.ccr.2006.10.012 17157788

[45] Wiznerowicz M Trono D. Conditional suppression of cellular genes: lentivirus vector-mediated drug-inducible RNA interference. J Virol (2003) 77:8957–61. doi: 10.1128/jvi.77.16.8957-8951.2003 PMC167245 12885912

[46] Goeman F Manni I Artuso S Ramachandran B Toietta G Bossi G. Molecular imaging of nuclear factor-y transcriptional activity maps proliferation sites in live animals. Mol Biol Cell (2012) 23:1467–74. doi: 10.1091/mbc.E12-01-0039 PMC3327325 22379106

[47] Chan CJ Andrews DM Smyth MJ. Receptors that interact with nectin and nectin-like proteins in the immunosurveillance and immunotherapy of cancer. Curr Opin Immunol (2012) 24:246–51. doi: 10.1016/j.coi.2012.01.009 22285893

[48] Samanta D Ramagopal UA Rubinstein R Vigdorovich V Nathenson SG Almo SC. Structure of nectin-2 reveals determinants of homophilic and heterophilic interactions that control cell-cell adhesion. Proc Natl Acad Sci USA (2012) 109:14836–40. doi: 10.1073/pnas.1212912109 PMC3443150 22927415

[49] Xu Y Zhou L Zong J Ye Y Chen G Chen Y. Decreased expression of the NKG2D ligand ULBP4 may be an indicator of poor prognosis in patients with nasopharyngeal carcinoma. Oncotarget (2017) 8:42007–19. doi: 10.18632/oncotarget.14917 PMC5522045 28159927

[50] Bottino C Castriconi R Pende D Rivera P Nanni M Carnemolla B. Identification of PVR (CD155) and nectin-2 (CD112) as cell surface ligands for the human DNAM-1 (CD226) activating molecule. J Exp Med (2003) 198:557–67. doi: 10.1084/jem.20030788 PMC2194180 12913096

[51] Chiossone L Chaix J Fuseri N Roth C Vivier E Walzer T. Maturation of mouse NK cells is a 4-stage developmental program. Blood (2009) 113:5488–96. doi: 10.1182/blood-2008-10-187179 19234143

[52] Cheng Q Xia J Wang K Zhang Y Chen Y Zhong Q. CRISPR/Cas9 ribonucleoprotein (RNP) complex enables higher viability of transfected cells in genome editing of acute myeloid cells. Ann Transl Med (2022) 10:862–22. doi: 10.21037/atm-22-3279 PMC9469150 36111017

