# Supplementary figures and images for: AATF/Che-1 RNA polymerase II binding protein overexpression reduces the anti-tumor NK-cell cytotoxicity through activating receptors modulation
Source: Front Immunol. 2023 Jun 26;14:1191908. doi: 10.3389/fimmu.2023.1191908 (PMC10332273; doi:10.3389/fimmu.2023.1191908)

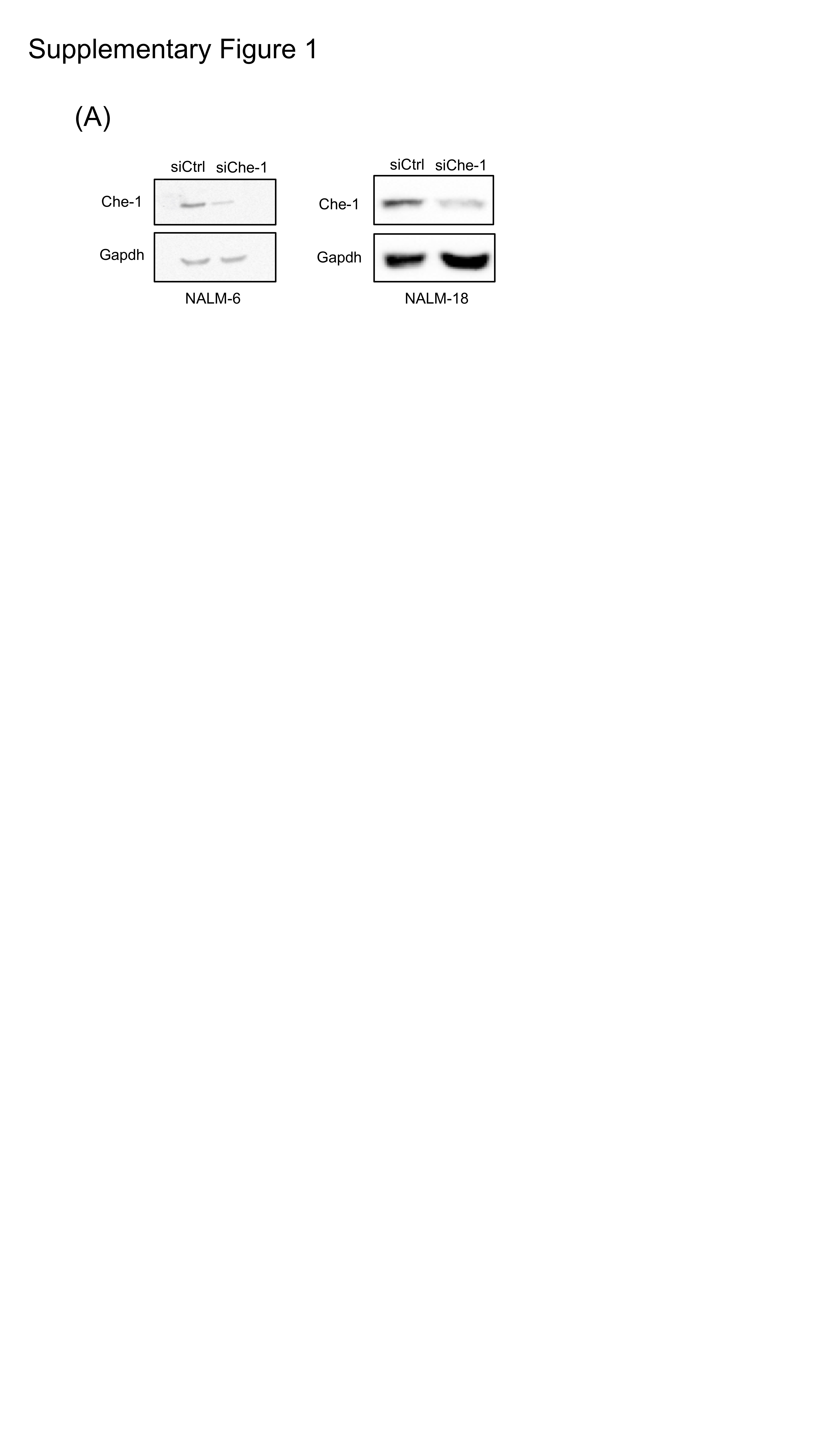

Supplement: Supplementary Figure 1 — Che-1 depletion by lentiviral transduction. (A) WB analysis of NALM-6 and NALM-18 cell lines transduced with siCtrl or siChe-1 sequences-carrying lentiviral plasmids to inhibit Che-1 expression. Gapdh antibody was used as loading control. [file Image_1.jpeg]

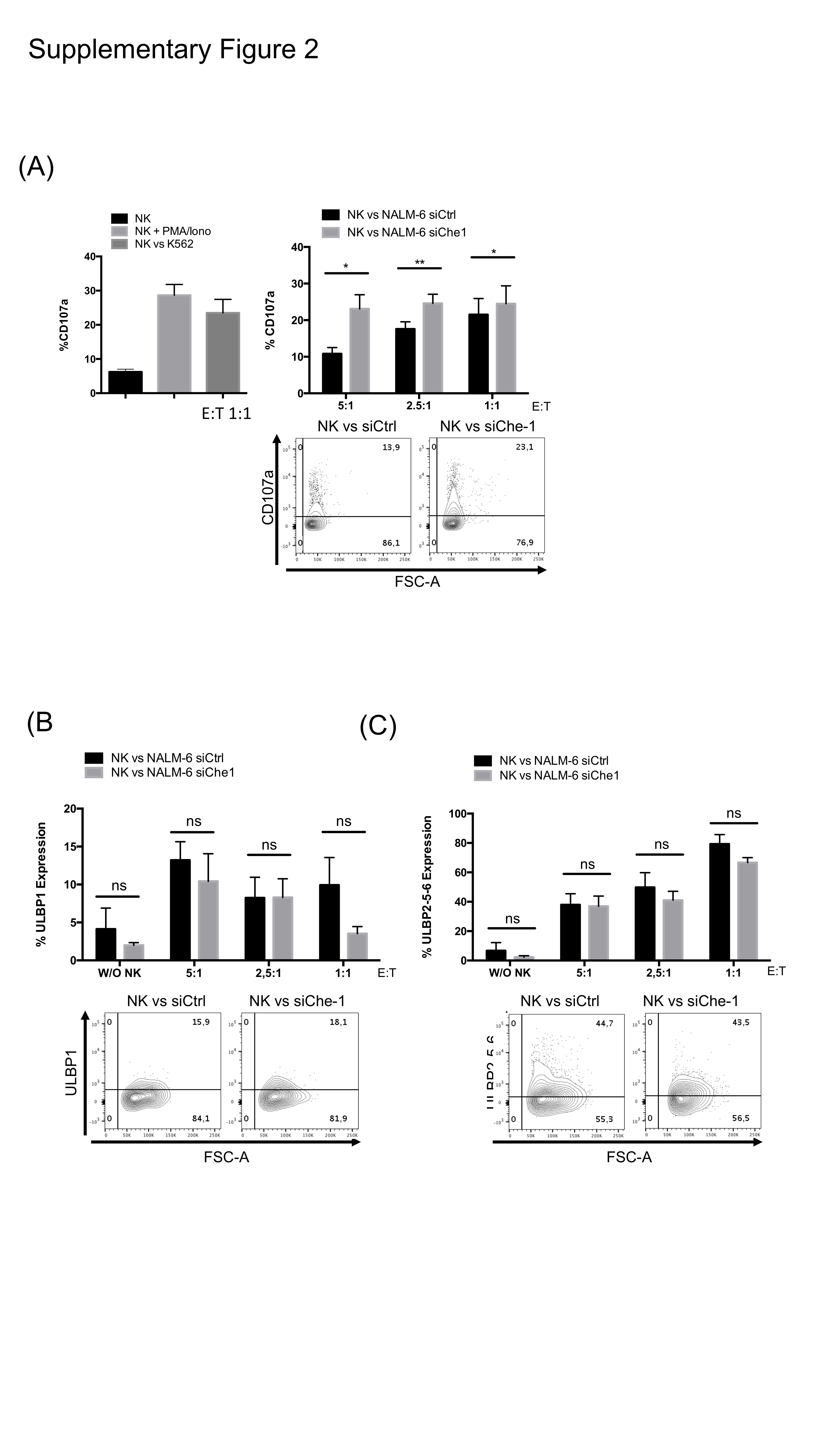

Supplement: Supplementary Figure 2 — siChe-1-dependent CD107a and ULBP family members modulation. (A) CD107a expression of NK-cells (CD19-/GFP-) by flow cytometry after a 4-hours-culture with NALM-6 siCtrl or siChe-1 at different Effector : Target (E:T) ratio (n=3). One Representative plot of CD107a expression of 3 performe. NK alone, Nk-cells stimulated with PMA/Ionomycyn and co-cultured were used as control cinditions (Left) (B Graph: flow-cytometry of ULBP1 expression of NALM-6 siCtrl and siChe-1 (CD19+/GFP+) after 16 hours of co-culture with NK-cells at different E:T ratio (n=3). One representative plot of ULBP1 expression out of 3 performed. Basal ULBP1 expression was measured in w/o NK cell condition (C) Graph: flow-cytometry of ULBP2-5-6 expression of NALM-6 siCtrl and siChe-1 (CD19+/GFP+) after 16-hour co-culture with NK-cells at different E:T ratio (n=3). One representative plot of ULBP2-5-6 expression out of 3 performed. Basal ULBP2-5-6 expression was measured in w/o NK cell condition. [file Image_2.jpeg]

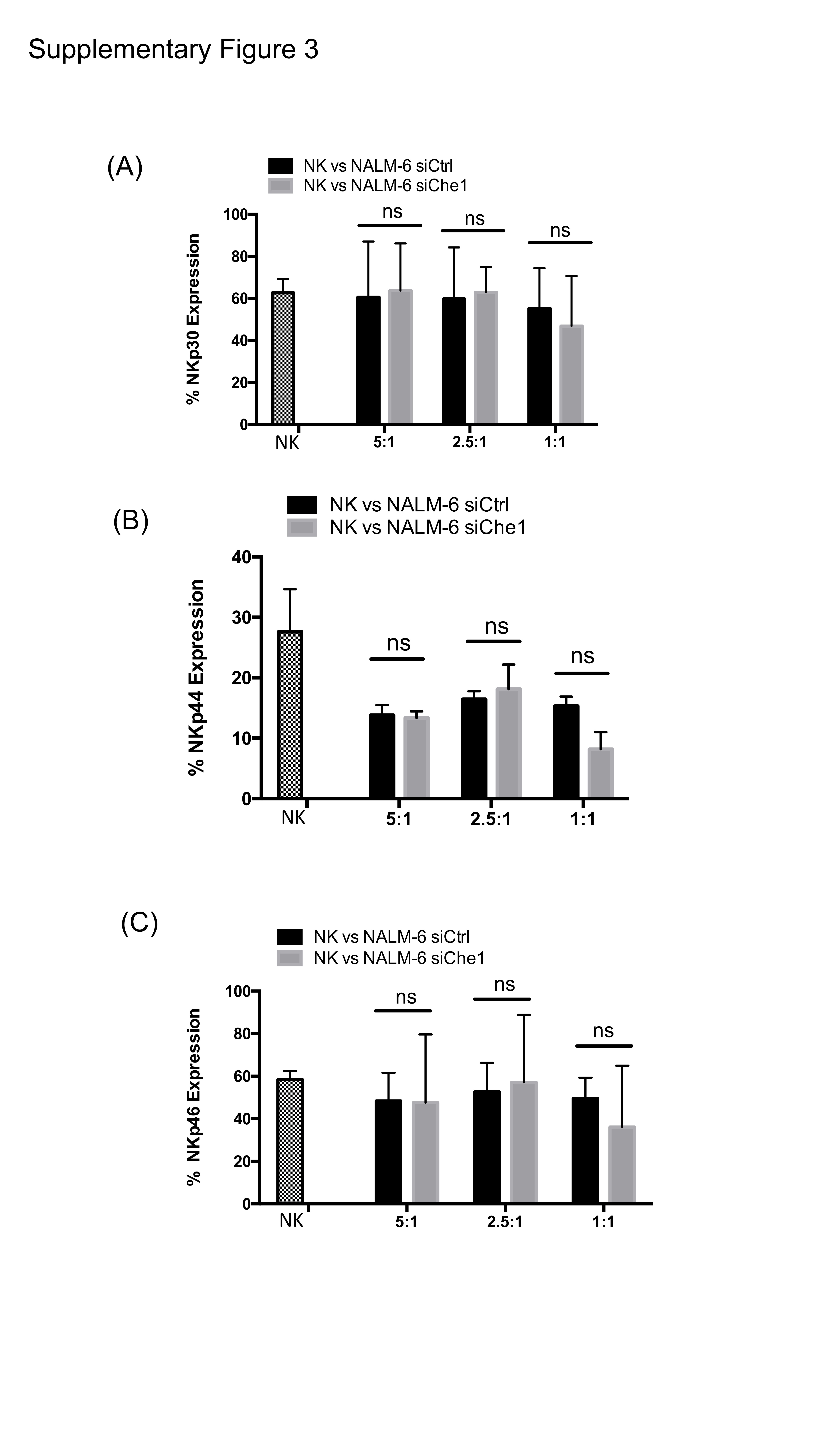

Supplement: Supplementary Figure 3 — Natural Citotoxicity Receptors (NCR) expression. (A) NKp30, (B) NKp44 and (C) NKp46 evaluation by flow cytometry of NK-cells(CD19-/GFP-) after a 16-hour co-culture with NALM-6 siCtrl and siChe-1 at different E:T ratio. [file Image_3.jpeg]
